# Low Sound Velocity Contributing to the High Thermoelectric Performance of Ag_8_SnSe_6_

**DOI:** 10.1002/advs.201600196

**Published:** 2016-07-07

**Authors:** Wen Li, Siqi Lin, Binghui Ge, Jiong Yang, Wenqing Zhang, Yanzhong Pei

**Affiliations:** ^1^ Key Laboratory of Advanced Civil Engineering Materials of Ministry of EducationSchool of Materials Science and EngineeringTongji University4800 Caoan RdShanghai201804China; ^2^ Beijing National Laboratory for Condensed Matter PhysicsInstitute of PhysicsChinese Academy of ScienceBeijing100190China; ^3^ Materials Genome InstituteShanghai UniversityShanghai200444China

**Keywords:** lattice thermal conductivity, sound velocity, thermoelectric

## Abstract

Conventional strategies for advancing thermoelectrics by minimizing the lattice thermal conductivity focus on phonon scattering for a short mean free path. Here, a design of slow phonon propagation as an effective approach for high‐performance thermoelectrics is shown. Taking Ag_8_SnSe_6_ as an example, which shows one of the lowest sound velocities among known thermoelectric semiconductors, the lattice thermal conductivity is found to be as low as 0.2 W m^−1^ K^−1^ in the entire temperature range. As a result, a peak thermoelectric figure of merit *zT* > 1.2 and an average *zT* as high as ≈0.8 are achieved in Nb‐doped materials, without relying on a high thermoelectric power factor. This work demonstrates not only a guiding principle of low sound velocity for minimal lattice thermal conductivity and therefore high *zT*, but also argyrodite compounds as promising thermoelectric materials with weak chemical bonds and heavy constituent elements.

## 1 Introduction

Thermoelectric (TE) energy conversion based on the Seebeck or Peltier effects, has been utilized for powering deep‐space missions or cooling sensitive electronics for decades.^1^ The efficiency of the thermoelectric material is determined by the dimensionless figure of merit, *zT = S* ^2^ *T*/*ρ*(*κ*_E_ + *κ*_L_), where *S* is the Seebeck coefficient, *ρ* is the electrical resistivity, *κ*_E_ and *κ*_L_ are the electronic and the lattice contribution to the thermal conductivity, and *T* is absolute temperature, respectively.

Due to the strongly coupled electrical properties between *S*, *ρ*, and *κ*_E_, simply tuning one of these parameters usually leads to a compensation in the others, resulting in the difficulty for enhancing *zT*. One successful strategy for improving *zT* is to enhance the power factor *S* ^2^/*ρ* through band engineering,^2^, ^3^, ^4^, ^5^, ^6^, ^7^ provided the carrier concentration is optimized.^8^ The other effective strategy is typified by minimizing the only one independent material property, the lattice thermal conductivity (*κ*_L_), through nanostructuring,^9^, ^10^, ^11^, ^12^, ^13^, ^14^, ^15^ liquid phonons,^16^, ^17^ and lattice anharmonicity.^18^, ^19^

Among the above strategies for lowering the lattice thermal conductivity, the commonness relies on the enhancement of the scattering on the phonons. It is well known that the lattice thermal conductivity (*κ*_L_) in a solid depends essentially on the heat capacity at a constant volume (*C*_v_), the sound velocity (*v*), and the mean free path (*l*) via *κ*_L_ = 1/3*C*_v_ *vl*. The above strategies for reducing *κ*_L_, put the emphasis on the phonon scattering for shortening the phonon mean free path. Alternatively, it can be seen as well that a low heat capacity should lead to a low *κ*_L_, however, there are very few demonstrated strategies to lower *C*_v_. This quantity can usually be approximated by the Dulong–Petit constant (≈3*k*_B_/atom, where *k*_B_ is the Boltzmann constant) for a Debye solid at high temperatures when thermoelectric power generation becomes important, leading to an approximation of *κ*_L_ = *k*_B_ *vl*.

This leaves an important approach in the community of thermoelectricity, but has been so far rarely emphasized on, for seeking a low lattice thermal conductivity in materials with a low sound velocity. Crystalline solids with a low sound velocity should ideally behave as “phonon‐glass electron‐crystal”^20^ thermoelectric materials. Many of the recent efforts have been taken to seek for new thermoelectrics of intrinsic ultra‐low thermal lattice thermal conductivity, which were usually ascribed to the complex crystal structure,^21^ large molecular weight,^22^, ^23^ or lattice distortion.^24^ The essentiality for the minimal lattice thermal conductivity and therefore the high thermoelectric performance, is partially due to the low sound velocity as well, resulting from its weak chemical bonds and heavy atomic mass of constituent elements.^25^ When the medium varies from a gas to a liquid and then to a solid, a sound wave travels faster and faster, which roughly indicates that a weakly bonded medium reduces its velocity. The sound velocities of the longitudinal (*v*_s_) and transverse (*v*_t_) waves propagating in a homogeneous 3D solid are, respectively, determined by the bulk (*B*) and shear (*G*) modulus via *v*_s_ = [(*B+*4*G*/3)/*d*]^0.5^ and *v*_t_ = (*G*/*d*)^0.^ ^5^, where *d* is the density.

The large family of the argyrodite compound with a common formula of Ag^I^_8_M^V^X^VI^_6_ (M = Si, Ge, Sn and X = S, Se, Te), have been wildly considered as ionic conductors.^26^ The existence of highly mobile silver ions suggests its weakly bonding to chalcogen anions.^27^ Most importantly, these compounds with heavy elements are found to have the lowest sound velocity (*v*) of 1000–1500 m s^−1^ among known thermoelectric semiconductors (*v* > 1500 m s^−1^) as surveyed in Table S1 (Supporting Information).

Experimentally, Ag^I^_8_M^V^X^VI^_6_ argyrodite semiconductors indeed show an ultra‐low lattice thermal conductivity of 0.2–0.3 W m^−1^ K^−1^ and therefore good thermoelectric performance without relying on outstanding electronic properties. For instance, the intrinsic compounds Ag_8_GeTe_6_ 28, 29 and Ag_8_SiTe_6_ 30 show a low lattice thermal conductivity, contributing to a *zT* of ≈0.5 at high temperatures. A further optimization on the carrier concentration leads to an increased *zT* of ≈0.8 in Ag_8_GeTe_6_.^31^

Guided by the above concept on low sound velocity for thermoelectrics, this work takes Ag_8_SnSe_6_ as an example, a material with the lowest sound velocity in known thermoelectric semiconductors and cheaper element Se as compared with the previously reported tellurides. Ag_8_SnSe_6_ shows a lattice thermal conductivity of only ≈0.2 W m^−1^ K^−1^, being one of the least thermally conductive dense solids.^18^, ^21^, ^30^, ^31^ In order to realize the high *zT* in this ultra‐low lattice thermal conductivity material, the carrier concentration is optimized through Nb‐doping on the Sn site without an expectation on a change in the sound velocity. As a result, a thermoelectric figure of merit, *zT* > 1.2 is achieved at high temperatures. Further due to the ultra‐low lattice thermal conductivity in the entire temperature studied, the average *zT* of ≈0.8 promotes this material as a superior candidate for power generation application at elevated temperatures. This work not only demonstrates the validity of low sound velocity for thermal insulators but also a new family of compounds with promising thermoelectric performance.

## 2 Result and Discussion

**Figure 1:**
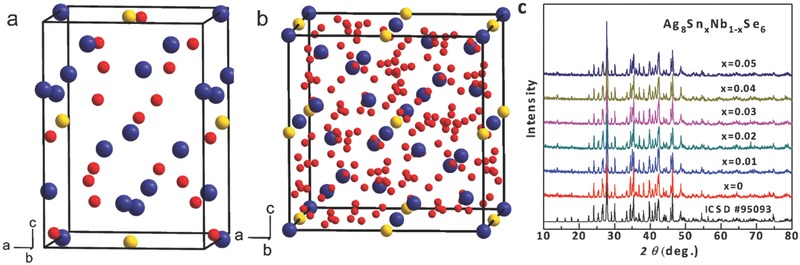
Crystal structure of a) orthorhombic β‐Ag_8_SnSe_6_ and b) cubic γ‐Ag_8_SnSe_6_. Red, yellow, and blue balls represent Ag, Sn, and Se atoms, respectively. c) X‐ray powder diffraction for Ag_8_Sn_1−_ *_x_*Nb*_x_*Se_6_ (*x* ≤ 0.05) shows no impurity peaks.

**Figure 2:**
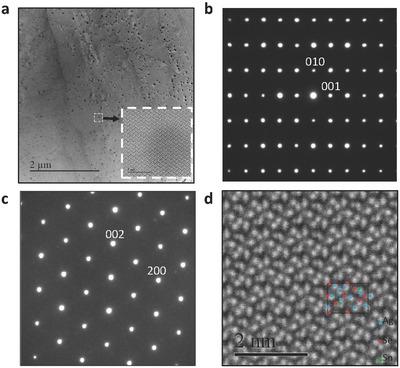
a) Low‐magnification TEM image with the corresponding diffraction patterns along b) [100] and c) [010] and d) high‐angle annular dark field (HAADF) image along [100] for Ag_8_SnSe_6_, revealing a single‐phase β‐Ag_8_SnSe_6_.

The detailed crystal structure of the Ag_8_SnSe_6_ has been well investigated in the literature.^32^ As shown in **Figure** **1**a,b, Ag_8_SnSe_6_ crystallizes either in a low‐temperature orthorhombic (Pmn21, β‐Ag_8_SnSe_6_) or a high‐temperature cubic (F4‐3m, γ‐Ag_8_SnSe_6_) structure. The X‐ray diffraction pattern (XRD) of the Ag_8_Sn_1−_ *_x_*Nb*_x_*Se_6_ samples (*x* ≤ 0.05) can be well indexed to the corresponding Pmn21 structure^32^ at room temperature, as shown in Figure 1c, indicating the high purity. The phase purity can be further confirmed by the transmission electron microscope (TEM) observations as shown in **Figure** **2**.

From the low‐magnification TEM image as shown in Figure 2a, uniformly distributed small precipitates in an average size of ≈5 nm (in dark) are observed. The volume concentration of these nanostructured precipitates is estimated statistically to be <2%. According to the energy‐dispersive X‐ray spectroscopy (EDS) mapping, the precipitates are enriched in Ag with an average Ag:Sn:Se mole ratio of 76.5:14:9.5. However, the high‐magnification TEM image indicates a negligible lattice distortion between the precipitates and the matrix, suggesting the same crystal structure for these precipitates. Moreover, the electron diffraction patterns (EDP) along both [100] (Figure 2b) and [010] (Figure 2c) directions indicate its single‐phase crystallized in the low‐temperature beta phase. The high‐angle annular dark field (HAADF) image (Figure 2d) is also in agreement with the crystal structure model of β‐Ag_8_SnSe_6_ projected along [100], where the blue, green, and red points in the model stand for Ag, Sn, and Se, respectively.

Nb‐doping at Sn site enables a Hall electron concentration varying from 10^16^ to 10^19^ cm^−3^, which is very helpful for understanding the transport properties for n‐type Ag_8_SnSe_6_. It is seen that Nb‐doping increases the Hall carrier concentration (*n*_H_), however a quantitative relationship between *x* and *n*_H_ is uneasy to estimate unfortunately. Because the carrier concentration quickly saturates at a very low value of <10^19^ cm^−3^ with Nb‐doping, a precise control of the carrier concentration is difficult. Since the transport properties directly depend on the carrier concentration, the room temperature Hall carrier concentration is used to identify the samples. It should be noted that the hysteresis on the transport properties is initially big but vanishes after a couple of thermal cycles.

**Figure 3:**
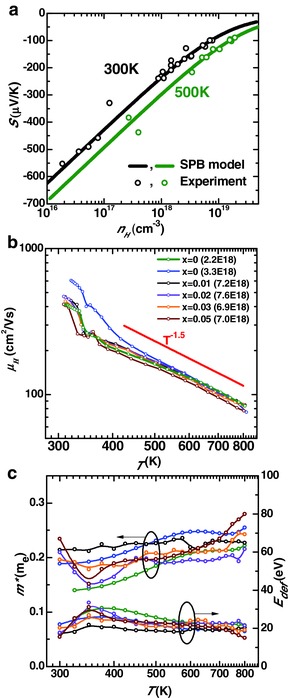
a) Hall carrier concentration–dependent Seebeck coefficient and b) temperature‐dependent hall mobility and c) density of state effective mass, deformation potential coefficient for Ag_8_Sn_1−_ *_x_*Nb*_x_*Se_6_ of different carrier concentration. The solid curves in (a) show the prediction based on the single parabolic band (SPB) model with a scattering mechanism by acoustic phonons.

As shown in **Figure** **3**a, the hall carrier concentration (*n*_H_) dependent Seebeck coefficient can be well predicted by a single parabolic band (SPB) model at both 300 and 500 K. The single band transport can be evidenced from the literature band structure calculation.^33^ The model assumes a carrier scattering by acoustic phonons, which is evidenced from the temperature (*T*) dependent Hall mobility (*μ*_H_) of *μ*_H_ ∼ *T* ^−1.5^ as shown in Figure 3b. It should be noted that the beta to gamma phase transition at ≈350 K affects the transport properties significantly. The single parabolic band model has shown great success on the prediction for thermoelectric transport properties in many thermoelectrics.^34^, ^35^, ^36^, ^37^, ^38^, ^39^

The single parabolic band model further enables an estimation on the density of states effective mass (m*) and the deformation potential coefficient (*E*_def_, scattering strength),^40^ which is shown in Figure 3c. Interestingly, both m* and *E*_def_ are found to be independent of temperature and doping concentration, indicating a rigid band behavior for Ag_8_Sn_1−_ *_x_*Nb*_x_*Se_6_. Not only an m* of 0.2 *m*_e_ (*m*_e_ is the free electron mass) but also an *E*_def_ of ≈25 eV falls in a typical range for thermoelectric semiconductors.^8^, ^35^, ^37^, ^41^, ^42^

**Figure 4:**
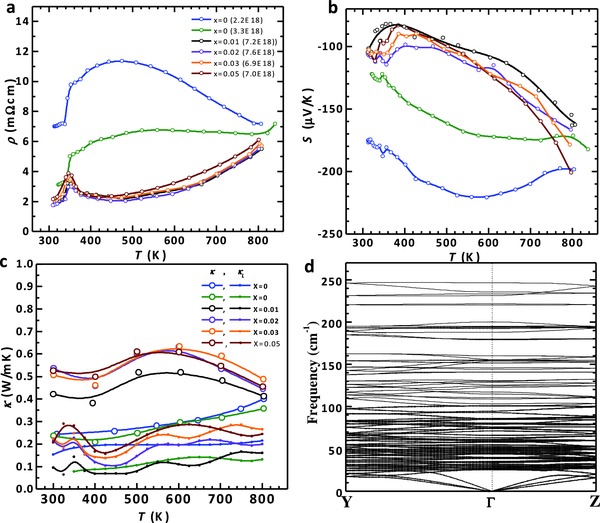
a) Temperature‐dependent resistivity, b) Seebeck coefficient, c) total thermal conductivity and lattice thermal conductivity for Ag_8_Sn_1−_ *_x_*Nb*_x_*Se_6_. d) The phonon dispersion relations for Ag_8_SnSe_6_ in the room temperature phase.

However, the Γ conduction band in Ag_8_SnSe_6_ comes with a valley degeneracy (*N*_v_) of only one,^33^ while conventional thermoelectrics have an *N*_v_ of 4 or higher.^2^, ^37^, ^43^, ^44^, ^45^ This should lead to its electronic performance not as promising as conventional thermoelectrics, which can be seen from the temperature‐dependent Seebeck coefficient and resistivity as shown in **Figure** **4**a,b, respectively. The resulting maximum thermoelectric power factor (*S* ^2^/*ρ*) of ≈6 μW cm^−1^ K^−2^ is much lower than 20–40 μW cm^−1^ K^−2^ that is normally seen in conventional thermoelectrics.^6^, ^10^, ^37^, ^45^, ^46^, ^47^, ^48^

Most interestingly, the thermal conductivity (*κ*) and its lattice conductivity (*κ*_L_) are found to be extremely low as shown in Figure 4c. *κ*_L_ is determined by subtracting the electronic contribution (*κ*_E_ = *LT*/*ρ*) from *κ*, with the Lorenz factor (*L*) is determined by the SPB model. Similar to many argyrodites reported so far,^28^, ^29^, ^30^, ^31^, ^49^ the observed *κ*_L_ of ≈0.2 W m^−1^ K^−1^ in the entire temperature range, is one of the lowest for dense bulk solids. Moreover, in the presence of Nb‐doping, the sound velocity of Ag_8_Sn_1−_ *_x_*Nb*_x_*Se_6_ (Table S1, Supporting Information) shows no observable difference, which is reasonable since the crystal structure and the composition do not change much. The *κ*_L_ ranging from 0.2 to 0.4 W m^−1^ K^−1^ at 600 K could possibly result from the uncertainties due to the total thermal conductivity (*κ*) measurement and the estimation of its electronic component (*κ*_E_), because the *κ* is extremely low (<0.6 W m^−1^ K^−1^) and largely (≈50%) comes from *κ*_E_. The most important is that the absolute value of the lattice thermal conductivity (*κ*_L_) is extremely low for all the samples due to the low sound velocity, further leading to a *zT* as high as 1.1 in Ag_8_SnSe_6_ without Nb‐doping.

It should be noted that argyrodite compounds usually undergo phase transitions, and most of the available sound velocities are measured at room temperature only. The commonality for this family of compounds is the similarly low values of both the sound velocity and the lattice thermal conductivity, regardless the crystal structures or the compositions. Although the sound velocity measurement is inaccessible here at temperatures other than room temperature, it should be similarly low for Ag_8_SnSe_6_ in both high and low‐temperature phases since the lattice thermal conductivity does not show any observable anomaly due to the phase transition. A statistical sound velocity measurement in this work, based on ten different Ag_8_Sn_1−_ *_x_*Nb*_x_*Se_6_ samples at room temperature, shows an average value of 1522 m s^−1^, which is lower than that in the literature.^50^ The standard deviation of this measurement is 1.4%, and the deviation between the measurement by the technique used here and that from literatures for a few different thermoelectric materials is <13% (Table S1, Supporting Information).

To shed light on the possible origin of the extremely low lattice thermal conductivity of Ag_8_SnSe_6_, we carried out the phonon dispersion calculation for room temperature phase as shown in Figure 4d. The most important feature relating to its extraordinary *κ*_L_ is the low sound velocities for the acoustic phonons. The theoretical sound velocities are 1189, 1318, and 2524 m s^−1^ for the two transverse and one longitudinal acoustic phonons, respectively. The averaged sound velocity is 1400 m s^−1^, which is among the lowest values of all known good thermoelectric materials (Table S1, Supporting Information). This can be traced back to the overall weak chemical bonds due to the high content of the weakly bonded monovalent Ag ions, similar to other superionic compounds with group IB elements.^51^, ^52^ These Ag ions also introduce low‐frequency optical phonons. Taking the lowest frequency optical phonon (25 cm^−1^) at Γ point for an example, its eigenvector clearly shows the localized vibrations from multiple Ag ions. This is just one typical optical phonon induced by Ag, and these dispersionless optical phonons are densely distributed from 20 to 70 cm^−1^ (Figure 4d). These optical phonons barely contribute to the phonon transport due to their very low sound velocities. On the other hand, the scattering channel of the phonon–phonon interaction is greatly enhanced,^53^, ^54^ leading to an additional *κ*_L_ reduction mechanism as reported previously.^17^ It is thus conclusive that the low sound velocity, together with the low frequencies of the optical phonons, are responsible for the low *κ*_L_ of Ag_8_SnSe_6_.

**Figure 5:**
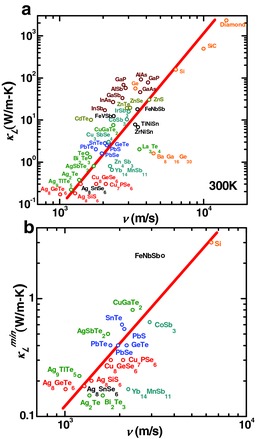
a) Sound velocity versus room temperature lattice thermal conductivity and b) the minimal lattice thermal conductivity measured experimentally for well‐studied thermoelectric semiconductors.

It is commonly believed that a high sound velocity fundamentally leads to a high lattice thermal conductivity according to *κ*_L_ = 1/3*C*_v_ *vl*. This can be clearly seen from the sound velocity dependent lattice thermal conductivity at room temperature for known semiconductors as surveyed in **Figure** **5**a and Table S1 (Supporting Information). However, the lattice thermal conductivity depends on the mean free path (*l*) as well, which is determined by the phonon scattering. This complicates the discussion on how sound velocity affects *κ*_L_ directly. Assuming that a maximal phonon scattering (i.e., minimal *l*) has been achieved for different materials, the resulting lattice thermal conductivity should be closely proportional to the sound velocity. This can be evidenced from Figure 5b, where the relationship between the sound velocity and the minimal lattice thermal conductivity measured experimentally (*κ*_L_^min^) is given for current well‐studied thermoelectrics. In this way, one can see a clear guiding principle of low sound velocity for minimal lattice thermal conductivity in practical thermoelectrics with a broad range of bonding stiffness.

**Figure 6:**
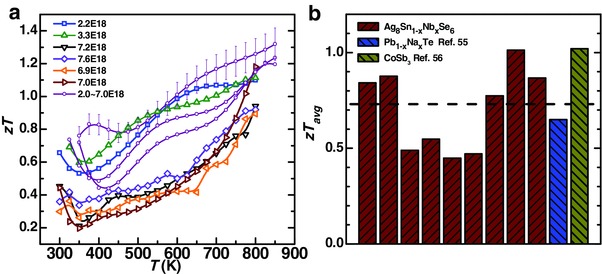
a) Temperature‐dependent figure of merit *zT* with a measurement error bar of 15% and b) the averaged *zT* for Ag_8_Sn_1−_ *_x_*Nb*_x_*Se_6_. The dashed line indicates the statistic average *zT*.

Unlike conventional thermoelectrics that usually require a high power factor,^6^, ^10^, ^37^, ^38^, ^39^, ^40^ Ag_8_Sn_1−_ *_x_*Nb*_x_*Se_6_ studied here presents a superior *zT* of 1.2 or higher, solely relying on an extremely low lattice thermal conductivity due to its minimal sound velocity. The temperature‐dependent figure of merit *zT* is shown in **Figure** **6**a. These samples not only exhibit high peak *zT* but also a high average *zT* in the whole temperature range. The statistic average *zT* over the entire temperature range, for nine samples with different carrier concentrations (1–7 × 10^18^ cm^−3^), reaches ≈0.8, which is quite comparable with conventional thermoelectric PbTe^55^ and CoSb_3_ 56 (Figure 6b).

## 3 Summary

In summary, a family member of semiconducting argyrodites, Ag_8_SnSe_6_ with carrier concentration in a broad range is studied. This exemplary material shows an extremely low lattice thermal conductivity of ≈0.2 W m^−1^ K^−1^ in the entire temperature, due to the low sound velocity. Without relying on a high power factor, which is usually required in conventional thermoelectrics, a peak *zT* of >1.2 and an average *zT* of ≈0.8 are still achievable in Ag_8_SnSe_6_, not only suggesting argyrodites as promising thermoelectrics but also demonstrating a strategy of low sound velocity for thermoelectrics in general.

## 4 Experimental Section

Polycrystalline samples of Ag_8_SnSe_6_ were synthesized by melting the stoichiometric amount of high purity elements (>99.99%) at 1173 K for 6 h, quenching in cold water and annealing at 900 K for 3 d. In this study, various dopants including niobium (Nb), vanadium (V), zinc (Zn), cadmium (Cd), antimony (Sb), and bismuth (Bi) were used to tune the carrier concentration. It is found that Nb‐doping is the most effective dopant, presumably due to its strong tendency to be pentavalent and the most comparable size between Nb^5+^ and Sn^4+^. Therefore, this work focuses on Nb‐doping with the stoichiometric composition of Ag_8_Sn_1−_ *_x_*Nb*_x_*Se_6_ (*x* ≤ 0.05). The obtained ingots were hand ground into fine powder for hot pressing at 900 K for 30 min under a uniaxial pressure of ≈65 MPa. The pellet samples for measurements were ≈12.0 mm in diameter and ≈1.5 mm in thickness and the density (*d*) is higher than 96% of theoretical value.

The details on the microstructural characterizations including powder X‐ray diffraction and (scanning) transmission electron microscope [(S)TEM], the measurements of the transport properties including resistivity, Seebeck coefficient, Hall coefficient, and thermal conductivity were given elsewhere.^5^, ^7^ Measurements of sound velocities were carried out on the pellet samples at room temperature. Longitudinal and transverse sound velocities were measured using pulse‐receiver (Olympus‐NDT) equipped with an oscilloscope (Keysight). Shear gel (Olympus) and water were used as couplants between the sample and the ultrasonic transducers for transverse and longitudinal sound velocity measurements, respectively.

Lattice dynamics for Ag_8_SnSe_6_ was investigated with the frozen phonon method^57^ which was implemented in the Phonopy package.^58^ The structure of Ag_8_SnSe_6_ is the low‐temperature phase with all the atoms ordered. The initial structures and atomic positions are taken from the reference.^32^ A 2 × 2 × 2 supercell (with 240 atoms in total) of the fully relaxed formula unit was constructed, and the Hellmann–Feynman forces for the supercells with small displacements (2 pm for each nonequivalent atom) were calculated. The k‐mesh was 6 × 6 × 4 for unit cell, and 3 × 3 × 2 for supercells with displacements. First‐principles calculations were performed within the Vienna ab initio simulation package (VASP).^59^ Generalized gradient approximation (GGA) functional and projected augmented wave (PAW) methods^60^, ^61^ were used. The convergence criteria were set to be 10^−4^ eV Å^−1^ for structural relaxation of the unit cell, and 10^−7^ eV for static calculations of displaced supercell to ensure the accuracy of phonon results. The plane wave cutoff was 300 eV. After the phonon dispersion was obtained, the sound velocity for each acoustic phonon mode was calculated by averaging the zone‐center velocities along different directions.

## Supporting information

As a service to our authors and readers, this journal provides supporting information supplied by the authors. Such materials are peer reviewed and may be re‐organized for online delivery, but are not copy‐edited or typeset. Technical support issues arising from supporting information (other than missing files) should be addressed to the authors.

Supplementary

## References

[1] L. E. Bell , Science 2008, 321, 1457. 18787160 10.1126/science.1158899

[2] Y. Pei , X. Shi , A. LaLonde , H. Wang , L. Chen , G. J. Snyder , Nature 2011, 473, 66. 21544143 10.1038/nature09996

[3] Y. Pei , H. Wang , Z. M. Gibbs , A. D. LaLonde , G. J. Snyder , NPG Asia Mater. 2012, 4, e28.

[4] W. Liu , X. Tan , K. Yin , H. Liu , X. Tang , J. Shi , Q. Zhang , C. Uher , Phys. Rev. Lett. 2012, 108, 166601. 22680741 10.1103/PhysRevLett.108.166601

[5] W. Li , Z. Chen , S. Lin , Y. Chang , B. Ge , Y. Chen , Y. Pei , J. Materiom. 2015, 1, 307.

[6] C. Fu , S. Bai , Y. Liu , Y. Tang , L. Chen , X. Zhao , T. Zhu , Nat. Commun. 2015, 6, 8144. 26330371 10.1038/ncomms9144 PMC4569725

[7] S. Lin , W. Li , Z. Chen , J. Shen , B. Ge , Y. Pei , Nat. Commun. 2016, 7, 10287. 26751919 10.1038/ncomms10287 PMC4729895

[8] Y. Pei , Z. M. Gibbs , B. Balke , W. G. Zeier , G. J. Snyder , Adv. Energy Mater. 2014, 4, 1400486.

[9] K. F. Hsu , S. Loo , F. Guo , W. Chen , J. S. Dyck , C. Uher , T. Hogan , E. K. Polychroniadis , M. G. Kanatzidis , Science 2004, 303, 818. 14764873 10.1126/science.1092963

[10] K. Biswas , J. He , I. D. Blum , C.‐I. Wu , T. P. Hogan , D. N. Seidman , V. P. Dravid , M. G. Kanatzidis , Nature 2012, 489, 414. 22996556 10.1038/nature11439

[11] B. Poudel , Q. Hao , Y. Ma , Y. C. Lan , A. Minnich , B. Yu , X. A. Yan , D. Z. Wang , A. Muto , D. Vashaee , X. Y. Chen , J. M. Liu , M. S. Dresselhaus , G. Chen , Z. F. Ren , Science 2008, 320, 634. 18356488 10.1126/science.1156446

[12] Y. Pei , J. Lensch‐Falk , E. S. Toberer , D. L. Medlin , G. J. Snyder , Adv. Funct. Mater. 2011, 21, 241.

[13] S. I. Kim , K. H. Lee , H. A. Mun , H. S. Kim , S. W. Hwang , J. W. Roh , D. J. Yang , W. H. Shin , X. S. Li , Y. H. Lee , G. J. Snyder , S. W. Kim , Science 2015, 348, 109. 25838382 10.1126/science.aaa4166

[14] H. Wu , F. Zheng , D. Wu , Z.‐H. Ge , X. Liu , J. He , Nano Energy 2015, 13, 626.

[15] Q. Zhang , X. Ai , L. Wang , Y. Chang , W. Luo , W. Jiang , L. Chen , Adv. Funct. Mater. 2015, 25, 966.

[16] H. Liu , X. Shi , F. Xu , L. Zhang , W. Zhang , Nat. Mater. 2012, 11, 422. 22406814 10.1038/nmat3273

[17] W. Qiu , L. Xi , P. Wei , X. Ke , J. Yang , W. Zhang , Proc. Natl. Acad. Sci. USA 2014, 111, 15031. 25288751 10.1073/pnas.1410349111 PMC4210332

[18] L.‐D. Zhao , S.‐H. Lo , Y. Zhang , H. Sun , G. Tan , C. Uher , C. Wolverton , V. P. Dravid , M. G. Kanatzidis , Nature 2014, 508, 373. 24740068 10.1038/nature13184

[19] D. T. Morelli , V. Jovovic , J. P. Heremans , Phys. Rev. Lett. 2008, 101, 035901. 18764265 10.1103/PhysRevLett.101.035901

[20] G. A. Slack , in CRC Handbook of Thermoelectrics (Ed: Rowe D. M.), CRC Press, Boca Raton, FL 1995, ch. 34, p. 406.

[21] K. Kurosaki , A. Kosuga , H. Muta , M. Uno , S. Yamanaka , Appl. Phys. Lett. 2005, 87, 061919.

[22] M. Christensen , A. B. Abrahamsen , N. B. Christensen , F. Juranyi , N. H. Andersen , K. Lefmann , J. Andreasson , C. R. H. Bahl , B. B. Iversen , Nat. Mater. 2008, 7, 811. 18758454 10.1038/nmat2273

[23] S. R. Brown , S. M. Kauzlarich , F. Gascoin , G. J. Snyder , Chem. Mater. 2006, 18, 1873.

[24] T. Yamada , H. Yamane , H. Nagai , Adv. Mater. 2015, 27, 4708. 26175276 10.1002/adma.201501970

[25] F. Li , J. Li , L. Zhao , K. Xiang , Y. Liu , B. Zhang , Y. Lin , C. Nan , H. Zhu , Energy Environ. Sci. 2012, 5, 7188.

[26] W. Kuhs , R. Nitsche , K. Scheunemann , Mater. Res. Bull. 1979, 14, 241.

[27] I. S. Osypyshyn , N. V. Chekailo , V. I. I. Vsesoyuzn , presented in part at the Conference of the Chemistry, Physics and Technical Applications of Chalcogenides, 1988, 3, 282.

[28] M. Fujikane , K. Kurosaki , H. Muta , S. Yamanaka , J. Alloy Compd. 2005, 396, 280.

[29] A. Charoenphakdee , K. Kurosaki , H. Muta , M. Uno , S. Yamanaka , Phys. Status Solidi RRL 2008, 2, 65.

[30] A. Charoenphakdee , K. Kurosaki , H. Muta , M. Uno , S. Yamanaka , Jpn. J. Appl. Phys. 2009, 48, 011603.

[31] T. J. Zhu , S. N. Zhang , S. H. Yang , X. B. Zhao , Phys. Status Solidi RRL 2010, 4, 317.

[32] L. D. Gulaya , I. D. Olekseyukb , O. V. Parasyukb , Journal of alloys and compounds 2001, 339, 113.

[33] M. Kocher , A. Jain , S. P. Ong , G. Hautier , (https://www.materialsproject.org/materials/mp‐17984/).

[34] A. F. May , E. S. Toberer , A. Saramat , G. J. Snyder , Phys. Rev. B 2009, 80, 125205.

[35] J. Shen , Z. Chen , S. Lin , L. Zheng , W. Li , Y. Pei , J. Mater. Chem. C 2016, 4, 209.

[36] H. Wang , Y. Pei , A. D. LaLonde , G. J. Snyder , Adv. Mater. 2011, 23, 1366. 21400597 10.1002/adma.201004200

[37] A. D. LaLonde , Y. Pei , G. J. Snyder , Energy Environ Sci. 2011, 4, 2090.

[38] Y. Pei , A. F. May , G. J. Snyder , Adv. Energy Mater. 2011, 1, 291.

[39] H. Xie , H. Wang , Y. Pei , C. Fu , X. Liu , G. J. Snyder , X. Zhao , T. Zhu , Adv. Funct. Mater. 2013, 23, 5123.

[40] J. Bardeen , W. Shockley , Phys. Rev. 1950, 80, 72.

[41] H. Wang , Y. Pei , A. D. LaLonde , G. J. Snyder , Proc. Natl. Acad. Sci. USA 2012, 109, 9705. 22615358 10.1073/pnas.1111419109 PMC3382475

[42] H. Wang , E. Schechtel , Y. Pei , G. J. Snyder , Adv. Energy Mater. 2013, 3, 488.

[43] C. Fu , T. Zhu , Y. Pei , H. Xie , H. Wang , G. J. Snyder , Y. Liu , Y. Liu , Z. Xinbing , Adv. Energy Mater., 2014, 4, 1400600.

[44] Y. Tang , Z. M. Gibbs , L. A. Agapito , G. Li , H. S. Kim , M. B. Nardelli , S. Curtarolo , G. J. Snyder , Nat. Mater. 2015, 14, 1223. 26436339 10.1038/nmat4430

[45] H. Wang , Z. M. Gibbs , Y. Takagiwa , G. J. Snyder , Energy Environ. Sci. 2014, 7, 804.

[46] L. Zhao , G. Tan , S. Hao , J. He , Y. Pei , H. Chi , H. Wang , S. Gong , H. Xu , V. P. Dravid , C. Uher , G. J. Snyder , C. Wolverton , M. G. Kanatzidis , Science 2016, 351, 141. 26612831 10.1126/science.aad3749

[47] Y. Gelbstein , J. Davidow , S. N. Girard , D. Y. Chung , M. Kanatzidis , Adv. Energy Mater. 2013, 3, 815.

[48] Z. Jian , Z. Chen , W. Li , J. Yang , W. Zhang , Y. Pei , J. Mater. Chem. C 2015, 3, 12410.

[49] K. S. Weldert , W. G. Zeier , T. W. Day , M. Panthöfer , G. J. Snyder , W. Tremel , J. Am. Chem. Soc. 2014, 136, 12035. 25058352 10.1021/ja5056092

[50] L. Li , Y. Liu , J. Dai , A. Hong , M. Zeng , Z. Yan , J. Xu , D. Zhang , D. Shan , S. Liu , Z. Ren , J.‐M. Liu , J. Mater. Chem. C 2016, 4, 5806.

[51] Y. Pei , N. A. Heinz , G. J. Snyder , J. Mater. Chem. 2011, 21, 18256.

[52] H. Liu , X. Shi , F. Xu , L. Zhang , W. Zhang , L. Chen , Q. Li , C. Uher , T. Day , G. J. Snyder , Nat. Mater. 2012, 11, 422. 22406814 10.1038/nmat3273

[53] L. Lindsay , D. A. Broido , T. L. Reinecke , Phys. Rev. B 2013, 87, 165201. 10.1103/PhysRevLett.111.025901 23889420

[54] J. Yang , L. Xi , W. Qiu , L. Wu , X. Shi , L. Chen , J. Yang , W. Zhang , C. Uher , D. Singh , NPJ Comput. Mater. 2016, 2, 15015.

[55] Y. Pei , A. LaLonde , S. Iwanaga , G. J. Snyder , Energy Environ Sci. 2011, 4, 2085.

[56] X. Shi , J. Yang , J. R. Salvador , M. F. Chi , J. Y. Cho , H. Wang , S. Q. Bai , J. H. Yang , W. Q. Zhang , L. D. Chen , J. Am. Chem. Soc. 2011, 133, 7837. 21524125 10.1021/ja111199y

[57] R. M. Martin , Electronic Structure: Basic Theory and Practical Methods, Cambridge University Press, Cambridge 2004.

[58] A. Togo , F. Oba , I. Tanaka , Phys. Rev. B 2008, 78, 134106.

[59] G. Kresse , J. Furthmüller , Phys. Rev. B 1996, 54, 169. 10.1103/physrevb.54.11169 9984901

[60] G. Kresse , D. Joubert , Phys. Rev. B 1999, 59, 1758.

[61] P. E. Blöchl , Phys. Rev. B 1994, 50, 17953. 10.1103/physrevb.50.17953 9976227

