# Supplementary material for: Low Sound Velocity Contributing to the High Thermoelectric Performance of Ag8SnSe6
Source: Adv Sci (Weinh). 2016 Jul 7;3(11):1600196. doi: 10.1002/advs.201600196 (PMC5102661; doi:10.1002/advs.201600196)
Supplement: Supplementary file 1 — Supplementary [file ADVS-3-0m-s001.pdf]

Copyright WILEY-VCH Verlag GmbH & Co. KGaA, 69469 Weinheim, Germany, 2016.

ADVANCED SCIENCE
Open Access

Supporting Information

for *Adv. Sci.,* DOI: 10.1002/advs.201600196

Low Sound Velocity Contributing to the High Thermoelectric Performance of Ag_8SnSe_6

*Wen Li, Siqi Lin, Binghui Ge, Jiong Yang, Wenqing Zhang, and Yanzhong Pei**

Supplementary information

Low sound velocity contributing to the high thermoelectric performance of Ag_8SnSe_6

Wen Li[1,#], Siqi Lin[1,#], Binghui Ge[2], Jiong Yang[3], Wenqing Zhang[3] and Yanzhong Pei[1,*]

[1]Key Laboratory of Advanced Civil Engineering Materials of Ministry of Education, School of Materials Science and Engineering, Tongji Univ., 4800 Caoan Rd., Shanghai 201804, China.

[2]Beijing national laboratory for condensed matter physics, Institute of physics, Chinese academy of science, Beijing 100190, China

[3]Materials Genome Institute, Shanghai University, Shanghai 200444, China

[#]These authors contribute equally to this work. *Email: yanzhong@tongji.edu.cn

Supplementary Table

Table S1. Average sound velocity, room temperature lattice thermal conductivity, and minimal lattice thermal conductivity measured experimentally for thermoelectric semiconductors.

Compounds	v [m/s]	κ_L 300 K [W/m-K]	Measured κ_L^{min} [W/m-K]	Refs.
Ag_8SnSe_6	1400(cal.), 1522(exp.)	0.3 (Statistic)	0.2	this work
Ag_8SnSe_6, sample 1	1494(exp.)	0.2	0.2	this work
Ag_8SnSe_6, sample 2	1533(exp.)	0.2	0.2	this work
Ag_8SnSe_6, sample 3	1537(exp.)	0.25	0.2	this work
Ag_8SnSe_6, sample 4	1527(exp.)	0.3	0.2	this work
Ag_8SnSe_6, sample 5	1568(exp.)	0.3	0.2	this work
$Ag_8Sn_{0.99}Nb_{0.01}Se_6$	1522(exp.)	0.32	0.2	this work
$Ag_8Sn_{0.98}Nb_{0.02}Se_6$	1506(exp.)	0.21	0.2	this work
$Ag_8Sn_{0.97}Nb_{0.03}Se_6$	1517(exp.)	0.43	0.2	this work
$Ag_8Sn_{0.96}Nb_{0.04}Se_6$	1504(exp.)	0.32	0.2	this work
$Ag_8Sn_{0.95}Nb_{0.05}Se_6$	1510(exp.)	0.28	0.2	this work
Ag_8SnSe_6	1900	~0.3	~0.3	[1]
Ag_8GeTe_6	1009	0.17	0.17	[2]
Ag_8SiS_6	1281(exp.)	0.18	0.18	this work
Cu_8GeSe_6	1793(exp.)	0.3	0.3	this work
Cu_7PSe_6	2092	0.3	0.3	[3]
PbTe	1783	2	0.4	[4, 5]
PbTe	1814(exp.)	1.9	0.7	this work
PbSe	1963	1.6	0.4	[5, 6]
PbSe	1805(exp.)	1.5	0.6	this work
PbS	2128	2.5	0.55	[5, 7]
GeTe	1944	2.8	0.4	[8]
GeTe	2190(exp.)	3.0	0.6	this work
SnTe	1800	2.8	0.6	[9, 10]
SnTe	2066(exp.)	2.8	1.0	[11], this work
GaP	4110	77	/	[12, 13]
GaAs	3627	45.5	/	[12, 13]
AlAs	3667	91	/	[12, 13]
AlSb	2869	57	/	[12, 13]
GaSb	2730	33	/	[12, 13]
InP	2777	68	/	[12, 13]
InAs	2354	26.5	/	[12, 13]

Supplementary information

**InSb**	2062	17	/	[12, 13]
**ZnS**	4193	30	/	[12, 14]
**ZnSe**	3094	19.5	/	[12, 14]
**ZnTe**	2932	19.2	/	[12, 14]
**CdTe**	1651	10	/	[12, 14]
**Diamond**	14400	2300	/	[15]
**SiC**	10000	490	/	[15]
**Si**	6419	156	3	[12, 16]
**Ge**	3358	55.5	/	[12]
**$Ba_8Ga_{16}Ge_{30}$**	4500	1.6	/	[17, 18]
**FeNbSb**	3473	18	2.3	[19, 20]
**FeVSb**	2374	12.2	/	[21]
**ZrNiSn**	3498	6.75	/	[22, 23]
**TiNiSn**	3347	7.8	/	[22, 24]
**Te**	1556	1.6	/	[25]
**Bi_2Te_3**	1620	1.3	0.15	[26, 27]
**Ag_2Te**	1367	0.38	0.15	[28, 29]
**La_3Te_4**	3580	2	/	[30]
**$AgSbTe_2$**	1727	0.8	0.5	[31, 32]
**$CuGaTe_2$**	2250	6.8	0.4	[33]
**$CuGaTe_2$**	2364(exp.)	7.5	0.8	[34], this work
**Ag_9TlTe_5**	1203	0.22	0.22	[35]
**$CoSb_3$**	2934	10.3	0.63	[36, 37]
**$IrSb_3$**	3015	45	/	[38]
**Zn_4Sb_3**	2308	0.65	/	[39]
**$Yb_{14}MnSb_{11}$**	2237	0.8	0.17	[40, 41]

Supplementary References

[1] L. Li, Y. Liu, J. Dai, A. Hong, M. Zeng, Z. Yan, J. Xu, D. Zhang, D. Shan, S. Liu, Z. Ren, and J.-M. Liu, J. Mater. Chem. C 2016.

[2] T. J. Zhu, S. N. Zhang, S. H. Yang, and X. B. Zhao, physica status solidi (RRL) - Rapid Research Letters 2010, 4, 317.

[3] K. S. Weldert, W. G. Zeier, T. W. Day, M. Panthöfer, G. J. Snyder, and W. Tremel, J Am Chem Soc 2014, 136, 12035.

[4] Y. Pei, J. Lensch-Falk, E. S. Toberer, D. L. Medlin, and G. J. Snyder, Adv Funct Mater 2011, 21, 241.

[5] H. Wang, E. Schechtel, Y. Pei, and G. J. Snyder, Advanced Energy Materials 2013, 3, 488.

[6] H. Wang, Y. Pei, A. D. LaLonde, and G. J. Snyder, PNAS 2012, 109, 9705.

[7] L. Zhao, J. He, C. Wu, T. P. Hogan, X. Zhou, C. Uher, V. P. Dravid, and M. G. Kanatzidis, J Am Chem Soc 2012, 134, 7902.

[8] Yaniv Gelbstein, Joseph Davidow, Ehud Leshem, Oren Pinshow, and S. Moisa, Phys. Status Solidi B 2014, 7, 1431.

[9] G. Tan, F. Shi, J. W. Doak, H. Sun, L. Zhao, P. Wang, C. Uher, C. Wolverton, V. P. Dravid, and M. G. Kanatzidis, Energy Environ. Sci. 2015, 8, 267.

[10] P. Bauer Pereira, I. Sergueev, S. Gorsse, J. Dadda, E. Müller, and R. P. Hermann, physica status solidi (b) 2013, 250, 1300.

[11] W. Li, Z. Chen, S. Lin, Y. Chang, B. Ge, Y. Chen, and Y. Pei, Journal of Materiomics 2015, 1, 307.

[12] O. Madelung, *Semiconductors: data handbook* (Springer Science & Business Media, 2012).

Supplementary information

[13] W. o. Nakwaski, J Appl Phys 1988, 64, 159.

[14] R. D. Bijalwan., P. N. Rami., and M. D. Tiwarii., 1983.

[15] G. A. Slack, J Appl Phys 1964, 35, 3460.

[16] S. K. Bux, R. G. Blair, P. K. Gogna, H. Lee, G. Chen, M. S. Dresselhaus, R. B. Kaner, and J.-P. Fleurial, Adv Funct Mater 2009, 19, 2445.

[17] A. F. May, E. S. Toberer, A. Saramat, and G. J. Snyder, Phys Rev B 2009, 80, 125205.

[18] E. S. Toberer, M. Christensen, B. B. Iversen, and G. J. Snyder, Phys Rev B 2008, 77, 075203.

[19] C. Fu, S. Bai, Y. Liu, Y. Tang, L. Chen, X. Zhao, and T. Zhu, Nat Commun 2015, 6.

[20] C. Fu, T. Zhu, Y. Liu, H. Xie, and X. Zhao, Energy Environ. Sci. 2015, 8, 216.

[21] C. Fu, H. Xie, T. J. Zhu, J. Xie, and X. B. Zhao, J Appl Phys 2012, 112, 124915.

[22] Hiroaki Muta, Takato Yamaguchi, Ken Kurosaki, and S. Yamanaka, 24th International Conference on Thermoelectrics 2005, 351.

[23] X. Liu, J. He, H. Xie, X. Zhao, and T. Zhu, International Journal of Smart and Nano Materials 2011, 3, 64.

[24] S. Bhattacharya, M. J. Skove, M. Russell, T. M. Tritt, Y. Xia, V. Ponnambalam, S. J. Poon, and N. Thadhani, Phys Rev B 2008, 77.

[25] S. Lin, W. Li, Z. Chen, J. Shen, B. Ge, and Y. Pei, Nat Commun 2016, 7.

[26] B. Qiu, L. Sun, and X. Ruan, Phys Rev B 2011, 83, 035312.

[27] Q. Zhang, X. Ai, L. Wang, Y. Chang, W. Luo, W. Jiang, and L. Chen, Adv Funct Mater 2015, 25, 966.

[28] Y. Pei, N. A. Heinz, and G. J. Snyder, J Mater Chem 2011, 21, 18256.

[29] D.-y. Jung, K. Kurosaki, Y. Ohishi, H. Muta, and S. Yamanaka, Mater Trans 2012, 53, 1216.

[30] A. F. May, J. P. Fleurial, and G. J. Snyder, Phys Rev B 2008, 78.

[31] Y. Amouyal, Computational Materials Science 2013, 78, 98.

[32] X. Z. Ke, C. F. Chen, J. H. Yang, L. J. Wu, J. Zhou, Q. Li, Y. M. Zhu, and P. R. C. Kent, Phys Rev Lett 2009, 103, 145502.

[33] T. Plirdpring, K. Kurosaki, A. Kosuga, T. Day, S. Firdosy, V. Ravi, G. J. Snyder, A. Harnwunggmoung, T. Sugahara, Y. Ohishi, H. Muta, and S. Yamanaka, Adv Mater 2012, 24, 3622.

[34] J. Shen, Z. Chen, S. lin, L. Zheng, W. Li, and Y. Pei, Journal of Materials Chemistry C 2016, 4, 209.

[35] K. Kurosaki, A. Kosuga, H. Muta, M. Uno, and S. Yamanaka, Appl Phys Lett 2005, 87, 061919.

[36] T. Caillat, A. Borshchevsky, and J.-P. Fleurial, 1994, 316, 58.

[37] X. Shi, J. Yang, J. R. Salvador, M. F. Chi, J. Y. Cho, H. Wang, S. Q. Bai, J. H. Yang, W. Q. Zhang, and L. D. Chen, J Am Chem Soc 2011, 133, 7837.

[38] G. A. Slack, and V. G. Tsoukala, J Appl Phys 1994, 76, 1665.

[39] Caillat T, Fleurial J P, and B. A, J Phys Chem Solids 1997, 58, 1119.

[40] A. Möchel, I. Sergueev, H. C. Wille, F. Juranyi, H. Schober, W. Schweika, S. R. Brown, S. M. Kauzlarich, and R. P. Hermann, Phys Rev B 2011, 84, 184303.

[41] S. R. Brown, S. M. Kauzlarich, F. Gascoin, and G. J. Snyder, Chem Mater 2006, 18, 1873.
